# Surgical management of a locally advanced jejunal stromal tumor: A case report of a challenging condition

**DOI:** 10.1016/j.ijscr.2023.109155

**Published:** 2023-12-12

**Authors:** Anis Hasnaoui, Racem Trigui, Nizar Khedhiri, Imen Helal, Haithem Zaafouri, Anis Ben Maamer

**Affiliations:** a Faculty of Medicine of Tunis, Tunis El Manar University, Rue Djebal Lakhdar, 1006 Tunis, Tunisia; b Department of General Surgery, Menzel Bourguiba Hospital, Tunisia; c Faculty of Medicine of Tunis, Tunis El Manar University, Rue Djebal Lakhdar, 1006 Tunis, Tunisia; d Department of Pathology, Habib Thameur hospital, Tunisia

**Keywords:** Gastrointestinal stromal tumors, Jejunal neoplasms, Locally advanced, Protein kinase inhibitors, Case report

## Abstract

**Introduction and importance:**

Locally advanced jejunal stromal tumors stand as a captivating and relatively rare entity, garnering attention for several reasons. Their inaccessible location by conventional endoscopy poses a diagnostic challenge. Further, treatment decisions necessitate a multidisciplinary approach, compounded by the absence of high-level evidence studies.

**Case presentation:**

A 54-year-old patient was admitted to our surgical department with abdominal pain and chronic anemia. Abdominal CT imaging confirmed the presence of a non-metastatic sizable jejunal tumor. The patient underwent laparotomy, revealing a locally advanced jejunal tumor contracting the ileum and the ascending colon. A monobloc oncological resection was performed, followed by the restoration of digestive continuity. Anatomopathological analysis delineated a locally advanced Stromal Tumor with a high risk of recurrence. The patient underwent a course of tyrosine kinase inhibitors for 3 years, with no reported recurrence during the subsequent 3-year follow-up.

**Discussion:**

Locally advanced jejunal stromal tumors are rare. Most patients present with unspecific symptoms. Diagnosis remains challenging due to their intricate anatomical location. Decisions regarding management must be deliberated within a multidisciplinary framework, tailored to each patient's unique characteristics. While combined therapeutic modalities have demonstrated efficacy in recent studies, prudence is advised given the heightened incidence of both short and long-term complications.

**Conclusion:**

In the absence of randomized controlled trials, the management of locally advanced jejunal stromal tumors underscores the imperative of multidisciplinary collaboration in treatment deliberations. A wide, sometimes mutilating excision is only permissible if it is complete.

## Highlights

• Locally advanced jejunal stromal tumors represent an uncommon occurrence.
• We should weigh the merits of immediate surgery with extensive multi-organ resection against a combined therapy.
• Multi-visceral resection in locally advanced GISTs is feasible with reasonable rates of morbidity and mortality.
• The prospect of a wide, mutilating excision is only deemed justifiable if it ensures complete removal of the tumor.

## 1 Introduction

Gastrointestinal stromal tumors (GISTs) are relatively rare mesenchymal tumors derived from Cajal cells. These tumors exhibit an uncertain evolving nature along with a distinctive immunohistochemical profile [1]. While the stomach accounts for the most common location in the majority of studies, comprising 55 % of all diagnosed GISTs, 31 % are found in the small bowel, and less than 6 % originate from the colorectal region [1]. At the initial diagnosis, a significant 88 % of GIST cases are localized and amenable to surgical resection, with only a mere 1.6 % classified as locally advanced, and 10 % presenting with metastasis [2]. Locally advanced jejunal stromal tumors stand as a captivating and relatively rare entity, garnering attention for several reasons. Their inaccessible location by conventional endoscopy poses a diagnostic challenge. Further, given the rarity of locally advanced GISTs, no randomized controlled trials have emerged to guide the management of this particular entity.

This case report has been reported in line with the SCARE Criteria [3].

## 2 Case presentation

A 54-year-old patient, with no medical or surgical history, was referred to our outpatient clinic due to persistent abdominal pain and chronic gastrointestinal bleeding revealed by intermittent occurrences of dark stools. Over the past five years, the patient had reported discomfort in the right hemiabdomen, without notable alterations in bowel movements or a decline in overall health.

**Fig. 1:**
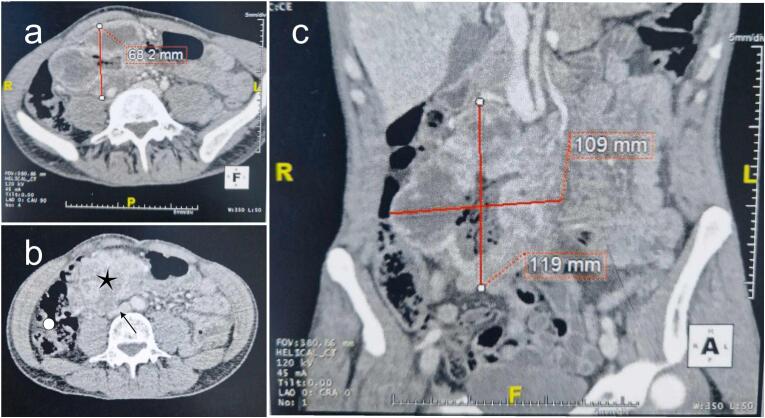
Preoperative computed tomography findings. (a), (b) axial views and (c) coronal view, showing a small bowel tumor (Black star in (b)), encompassing a necrotic region, and exerting pressure on the inferior vena cava (Black arrow in (b)) and the adjacent ascending colon (White circle in (b)).

Upon examination, the patient appeared pallid. Blood pressure was measured at 13/7 mmHg, and the heart rate at 90 beats per minute. Respiratory assessments fell within the normal range. An abdominal examination unveiled a solid, painless mass in the right hemiabdomen, measuring 14*10 cm, without signs of peritoneal inflammation. A rectal examination affirmed the presence of prolapsed hemorrhoids and normal-colored stools. Blood analyses disclosed regenerative chronic anemia with a hemoglobin level of 10 g/dl associated with low serum iron and ferritin levels. White blood cell and platelet counts exhibited normalcy, with no discernible impairments in renal function or electrolyte balance. A thoraco-abdominal CT scan, revealed a 12*11*7 cm heterogeneous small bowel tumor, encompassing a necrotic region, and exerting pressure on the inferior vena cava and the adjacent ascending colon (Fig. 1). No secondary locations were identified. Following a multidisciplinary hearing, the diagnosis of a resectable intestinal tumor was made, prompting the unanimous decision to pursue surgery as the primary course of action without the need of endoscopic exploration or preoperative biopsy.

**Fig. 2:**
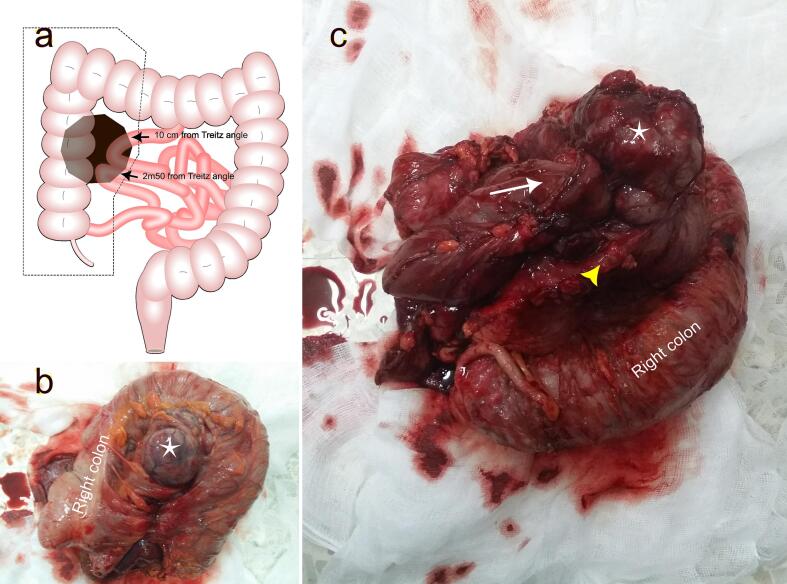
Intraoperative findings. (a) Illustration of the intraoperative findings. A locally advanced jejunal tumor contracting anr ileal loop positioned 2.5 m distal to the duodeno-jejunal flexure, and the ascending colon (Black transparent polygon). Surgical resection limits are highlighted with dashed lines. (b) Anterior view of the surgical specimen with the tumor highlighted with a white star. (c) Posterior view of the surgical specimen showing the tumor (White star) emerging from a jejunal loop (White arrow) and contracting an ileal loop (Yellow arrowhead) and the ascending colon. (For interpretation of the references to colour in this figure legend, the reader is referred to the web version of this article.)

The patient underwent a midline laparotomy. Exploration confirmed the absence of peritoneal nodules or palpable hepatic metastases and unveiled a substantial tumor originating from a jejunal loop located 10 cm distal to the duodeno-jejunal flexure, contracting an ileal loop positioned 2.5 m distal to the duodeno-jejunal flexure and 1 m from ileocecal valve, and the ascending colon. Given these findings, a monobloc oncological resection was executed, involving the removal of 15 cm of jejunum, 20 cm of ileum, and the ascending colon (Fig. 2). Immediate restoration of digestive continuity was performed as follows: An end-to-end jejuno-jejunal anastomosis, an end-to-end ileo-ileal anastomosis, and a side-to-side ileocolic anastomosis. The postoperative course was uneventful.

**Fig. 3:**
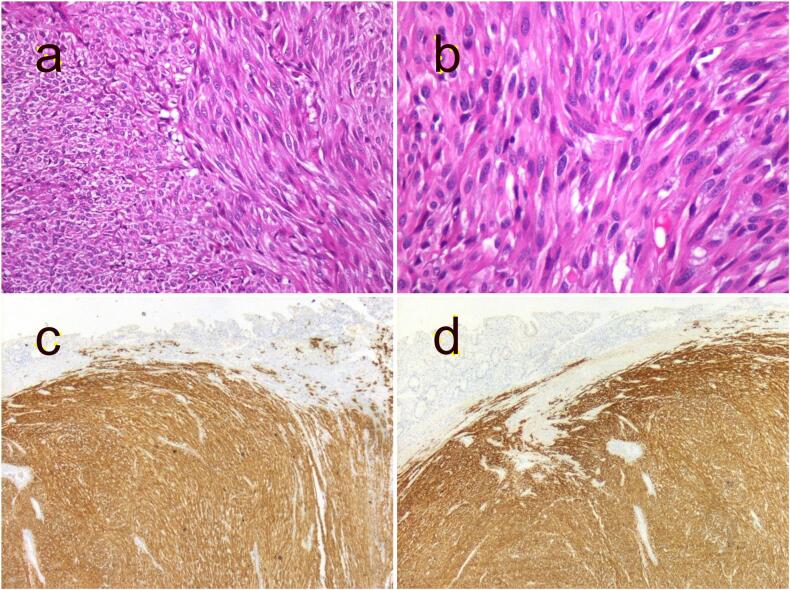
Histopathological findings of the jejunal stromal tumor. (a) Spindle cell GIST with closely packed elongated cells arranged in short intersecting fascicles (HEx200). (b) Elongated cells with blunt-ended cigar-shaped nuclei (HEx400). (c) CD117 (c-Kit) immunohistochemistry shows diffuse positivity. (d) Dog1 immunohistochemistry shows diffuse positivity.

The anatomopathological report confirmed the diagnosis of an intestinal stromal tumor, measuring 14*10 cm in size, with a mitotic count of 20/50 high-power fields. Immunohistochemistry demonstrated diffuse positivity for CD 117 and Dog 1 (Fig. 3). Surgical margins were microscopically negative for residual tumor cells. It was classified as a GIST with high relapse risk. Therefore, the patient received targeted therapy based on Imatinib for a duration of 3 years. Follow-up assessments revealed no recurrence of the tumor.

## 3 Discussion

Jejunal GISTs account for approximately 30 % of the overall spectrum of Gastrointestinal Stromal Tumors (GISTs) [1]. This subtype predominantly affects older male patients, with the median age at diagnosis standing at 60 years [4]. Patients are commonly referred with non-specific symptoms, with gastrointestinal bleeding being the most prevalent manifestation. Abdominal pain, small bowel obstruction, and bowel perforation are also reported symptoms [5]. Due to the non-specific clinical and imaging findings, and the challenges of accessing the tumor site using conventional endoscopy, arriving at a definitive diagnosis of jejunal GIST, prior to surgery, remains challenging [2]. In the case of our patient, given the inaccessibility of the tumor through standard endoscopic methods, and with double-balloon endoscopy and video capsule unavailable, a decision to proceed with potentially curative surgery based on CT scan findings was reached through a multidisciplinary hearing.

In the pre-imatinib era, the management of locally advanced jejunal GISTs was based on multi-visceral monobloc resection with negative margins [6]. Since 2012, given the morbidity and the risk of positive resection margins in multi-visceral resection, some authors have suggested using neoadjuvant tyrosine kinase inhibitors to shrink locally advanced tumors before undergoing surgery [6]. The management of locally advanced non-metastatic jejunal GISTs is challenging. While imatinib's effectiveness in treating metastatic GISTs is unquestionable, its use as a neoadjuvant approach remains controversial [7]. The main role of tyrosine kinase inhibitors in the neoadjuvant phase is to downsize locally advanced tumors, allowing surgical resection without recurring mutilating surgery [4,8]. Due to the effectiveness of this treatment, the latest guidelines issued by the European Society for Medical Oncology and the National Comprehensive Cancer Network recommended the use of imatinib as neoadjuvant therapy in locally advanced GISTs [4,8]. Although the association of tyrosine kinase inhibitors with surgery created a paradigm-shifting concept in managing locally advanced GISTs, this new line of treatment must be used with caution, as it was associated with high rates of postoperative complications and impaired anastomotic healing by aberrant tissue microstructure and inappropriate expressions of matrix metalloproteinase (MMP), Col1A1, and Col3A1 [9]. Further, a growing body of evidence highlights an array of debilitating side effects that can impede patients' overall prognosis. Among these adverse outcomes are muscle loss, anorexia, and dysphagia. These side effects will adversely affect the patient's short and long-term prognosis [10]. In addition, before prescribing imatinib in the neoadjuvant course for 6 to 12 months, the risks of major complications related to spontaneous evolution of the jejunal GIST (rupture, perforation, and hemorrhage) must be considered [[11], [12], [13]]. Finally, whereas locally advanced jejunal GISTs have shown similarities with their analogs in the stomach, clinical evolution appears to be less favorable [14,15].

On the other hand, studies comparing multi-organ resection alone vs combined therapy (Imatinib in the neoadjuvant phase followed by surgery) have shown that surgery with multi-visceral resection in locally advanced GISTs can be performed with reasonable rates of morbidity and mortality [16]. By exploring this multifaceted interplay between imatinib efficacy and its associated challenges, the aim of multidisciplinary hearings is to decide a course of action adherent to each patient characteristics. Full surgical excision of these malignancies is the only possible, efficient course of treatment. Surgery for jejunal GISTs must obey several standards, with the most essential is monobloc complete resection on a microscopic level. These tumors must be handled with extreme care. An intraoperative rupture must be avoided at all costs. It is an independent predictor of tumor recurrence despite complete macroscopic surgery [4,8]. The presence of GISTs in the jejunum is typically characterized by a minimal occurrence of lymph invasion and recurrence. As a result, there is no necessity for lymph node harvesting or systematic oncological resection when treating these small bowel tumors [12]. In our case, the jejunal GIST was locally advanced and we did not have histological proof of the tumor's nature. Therefore, a monobloc multi-visceral oncological resection was performed.

In early 2000, adjuvant therapy did not have much impact on managing jejunal GISTs as chemotherapy and radiation therapy proved ineffective [17]. It wasn't until 2009 that targeted therapy, based on Imatinib, revolutionized the GIST postoperative course. Three phase III trials have confirmed that patients with intermediate and high-risk of relapse, according to Miettinen or Joensuu classifications, have better survival without recurrence if imatinib is prescribed in the postoperative course [[18], [19], [20]]. In the reported case, the tumor was locally advanced and with high risk of relapse. Therefore, the patient was initiated systematically on imatinib for 3 years in the post-operative course.

## 4 Conclusion

Locally advanced jejunal GISTs represent an uncommon occurrence. Managing this condition presents a formidable challenge, compounded by the absence of randomized controlled trials to substantiate existing guidelines. Critical decisions are forged in multidisciplinary hearings, weighing the merits of immediate surgery with extensive multi-organ resection against a composite approach incorporating neoadjuvant therapy prior to surgical intervention. The prospect of a wide, occasionally mutilating excision is only deemed justifiable if it ensures complete removal of the tumor.

## Abbreviations

GISTs: Gastrointestinal stromal tumors

## Consent for publication

Written informed consent was obtained from the patient for publication and any accompanying images. A copy of the written consent is available for review by the Editor-in-Chief of this journal on request.

## Ethical approval

Ethical approval was deemed unnecessary by our institutional ethical committee, as the paper is reporting a single case that emerged during normal practice.

## Funding

Nothing to declare.

## Author contribution

Anis Hasnaoui: Conceptualization, Writing-Reviewing and Editing. Racem Trigui: writing-Original draft preparation. Nizar Khedhiri: Data curation. Imen Helal: Data curation. Haithem Zaafouri: Writing-Reviewing. Anis Ben Maamer: Writing-Reviewing. All authors read and approved the final manuscript.

## Guarantor

Anis Hasnaoui

## Conflict of interest statement

Nothing to declare.
